# Exploiting the Waste Biomass of Durian Shell as a Heterogeneous Catalyst for Biodiesel Production at Room Temperature

**DOI:** 10.3390/ijerph20031760

**Published:** 2023-01-18

**Authors:** Che Zhao, Hongyuan Chen, Xiao Wu, Rui Shan

**Affiliations:** 1 School of Naval Architecture and Maritime, Zhejiang Ocean University, Zhoushan 316022, China; 2 Guangzhou Institute of Energy Conversion, Chinese Academy of Sciences, Guangzhou 510640, China; 3 Fisheries College, Tianjin Agricultural University, Tianjin 300384, China

**Keywords:** waste biomass, durian shell, transesterification, heterogeneous, biodiesel

## Abstract

Durian shell, a biomass waste, was simply burned and then could serve as a heterogeneous catalyst for the transesterification reaction of palm oil with methanol at room temperature. The chemical composition, structure, and morphology of the catalyst were well-characterized by XRD, BET, SEM, TEM, EDS, TGA, FT-IR, and XPS measurement. With the preparation temperature rising to 350 °C, the maximum yield of the biodiesel could reach 94.1% at room temperature, and the optimum reaction conditions were 8 wt.% catalyst, 8:1 methanol/oil molar ratio, ad 2.5 h reaction time. The characterizations results indicated that K_2_O and K_2_CO_3_ existed on the surface of catalyst, and a moderate amount of carbon, which acts as a carrier, attributed to the activity of the catalyst. After repeating five times, the catalyst prepared at 350 °C showed better stability than other catalysts. This might be because the incomplete combustion of the remaining carbon slowed down the loss of K to some extent.

## 1. Introduction

With the rapid development of the world, resources such as oil are now suffering from a huge energy crisis due to their great exploitation and consumption [1]. According to figures released in BP’s 2017 Global Energy Statistics Report, in 2016, fossil energy such as coal, oil, and natural gas could take up at 85.5% of the total global energy consumption. The huge consumption of fossil fuels is followed by the further increase of environmental pollution caused by CO_2_ emissions [2,3]. Therefore, it is imperative to find suitable and alternative energy sources from the perspective of meeting global energy needs and mitigating environmental crisis [4,5].

While there are a certain number of substitutes of renewable energy that are likely to meet the energy needs of future society and reduce greenhouse gas emissions, biodiesel is considered as the most promising and easy to implement alternative to traditional diesel based on the existing infrastructure [5,6,7,8]. As a conventional, environment-friendly “green energy”, biodiesel has good starting performance, fuel properties, widely available raw materials, and renewability and can be used directly in existing diesel engines [9,10]. At present, homogeneous catalysts are widely used in the industrial production of biodiesel for transesterification between vegetable oil and methanol [11,12,13]. However, this process requires a variety of refining processes (such as acid neutralization), and the accompanied saponification reaction makes it difficult to separate the product from the mixture [14]. In addition, homogeneous catalysts will inevitably produce environmentally harmful wastewater during the recycling process [15,16]. Therefore, in recent years, as a potential choice, heterogeneous catalysts are easier to separate and can eliminate refining steps, which has led to widespread interest in them [17,18,19,20]. However, heterogeneous catalysts for biodiesel production synthesis are mostly chemically synthesized, which is contrary to the background of green economy and sustainable development, so the development and application of bio-derived catalysts is very promising [21].

The biomass including crop straw, forestry residues, agricultural product processing waste, livestock and poultry manure, and municipal solid waste abandoned by human society is the largest source of environmental pollution, with a total of more than 70% [22,23]. If not fully utilized, serious emissions problems will be formed [24]. According to statistics, there are currently 170 billion tons of biomass resources in the world, and China carries nearly one-third of the world’s low-grade biomass emissions [25]. Therefore, the high-value-added chemicals arising from waste biomass will need to be given high priority in solving the transformation and upgrading of the environment, energy, and materials [26,27].

In recent years, many researchers developed the direct calcine method for biodiesel production by using biomass as the raw catalyst, such as potassium- or calcium-rich materials [28,29,30]. These as-prepared catalysts could exhibit high catalytic activity even at room temperature. For example, Vadery et al. adapted coconut shell as a raw material to obtain potassium-rich ash through simple calcination and used it in the transesterification reaction for biodiesel. The results showed that there are many alkali metals and alkaline earth metals in the ash obtained from the simple calcination of coconut shell, which provided the active site for a transesterification reaction to produce biodiesel. Under the reaction temperature of 45 °C, 97% biodiesel can be obtained [31]. Meanwhile, Pathak et al. found that banana peel, after simple calcination, also had similar properties as coconut shell. The preparation of the catalyst is also carried out through simple steps: only through controlled heat treatment (calcination) without any chemical treatment. The obtained ash could be directly used for the preparation of biodiesel [32]. Sitepu et al. found that palm bunch ash contained potassium oxide as a major component that could catalyze palm oil to biodiesel at room temperature, with 98.9% biodiesel yield obtained [29]. The advantage of such catalysts is that their inherently high content of alkali metals or alkaline earth metal oxides could provide sufficient active substances at room temperature. However, these catalysts with high activity at room temperature still have the problem of alkali metals loss, and no more research has been done on the stability of these catalysts. The effect of catalytic activity and stability on the ratio of carbon to potassium in the ash has not been studied.

In our research, for the development of green synthesis methods, we synthesized a heterogeneous catalyst prepared by simply calcining durian shells, which enables the production of biodiesel at room temperature by transesterification of palm oil. The calcined temperature was investigated to examine the amount of carbon existing in the catalyst. Meanwhile, the physico/chemical properties of the samples were characterized. The reaction conditions, such as catalyst amount, methanol/oil molar ratio, reaction time, and reaction temperature at the catalyst activity, were investigated. Furthermore, the reusability of the catalyst was also discussed.

## 2. Experimental

### 2.1. Preparation and Collection of Catalyst

Durian shell was collected from the local farmer’s market and then washed completely by deionized water and then dried. Subsequently, the above-mentioned dried shell was crushed into small pieces with a blender, followed by thorough combustion in air, and was then milled to obtain an ash catalyst. The catalyst prepared under different conditions was named DN-n, where n is the treatment temperature of the durian shell. For comparison, the catalyst obtained by pyrolysis of the durian shell at 350 °C in a nitrogen atmosphere was named PDN-n.

### 2.2. Characterization of the Ash Catalyst

The surface morphology and physical properties of the catalyst samples were characterized by several techniques. Powder X-ray diffraction (XRD) was conducted to analyze the crystal phase of the material and the purity of the samples. Scanning electron microscopy (SEM) equipped with EDS detector was performed to observe the sample surface morphology and analyze elements of the samples. The internal morphology of the sample was observed by transmission electron microscopy (TEM). N_2_ adsorption method and the Brunauer–Emmett–Teller model (BET) were used on calculating specific surface area and pore structure. The Fourier transform–infrared spectra (FT-IR) of the samples were evaluated to investigate the surface functional groups in the 400–4000 cm^−1^ region. X-ray photoelectron spectroscopy (XPS) was carried out using a KRATOS XSAM800 (Kratos, Manchester, UK.), which was equipped with energy analyzers and monochromated. The thermogravimetric analysis (TGA) was performed by using a Stanton Redcroft STA-780 thermal analyzer between 30 and 850 °C.

### 2.3. Transesterification Reaction of Palm Oil

The transesterification reaction for the biodiesel preparation was carried out in a three-necked flask (200 mL) equipped with a condensing reflux unit, which was heated by a water bath. In one cycle, a certain ratio of catalyst (catalyst amount ranging from 3 wt.% to 8 wt.%) and methanol (molar oil molar ratio ranging from 3:1 to 9:1) was added into the palm oil (50 g). At a stirring speed of 600 rpm, the heating temperature was controlled at 20 to 70 °C, while the reaction time was controlled between 120 and 270 min. The catalyst was separated by filtration after reaction. The obtained biodiesel was analyzed by GC-MS to identify whether it met ASTM and EN standards after being separated from the product by a separating funnel.

### 2.4. Reusability Analysis

In order to further evaluate its performance, the reusability of the catalyst was carried out. After reaction, the catalyst was filtrated and washed by methanol and tetrahydrofuran for removing impurities and then dried in a vacuum drying oven under 90 °C for 6 h for the next cycle.

## 3. Results and Discussion

### 3.1. Catalyst Characterization

**Figure 1:**
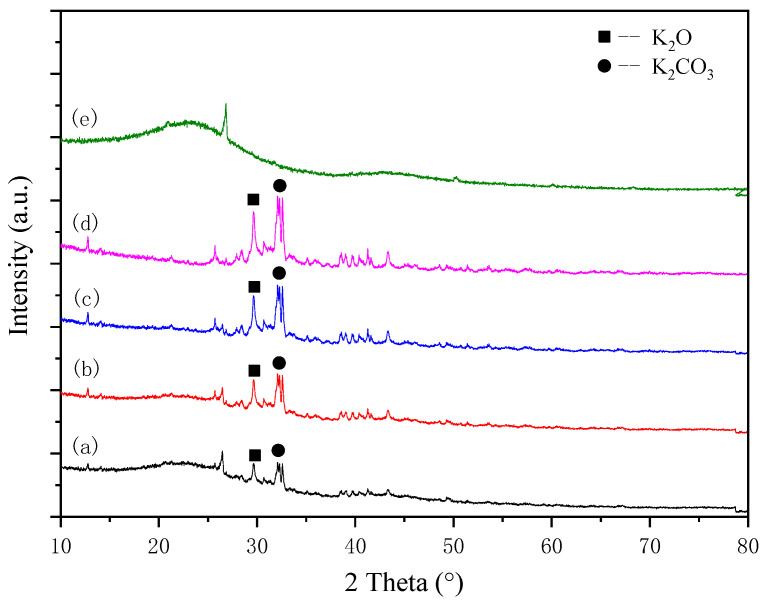
The XRD spectrum of the catalyst samples: (a) DN-250, (b) DN-300, (c) DN-350, (d) DN-400, and (e) PDN-350.

As shown in Figure 1, the XRD spectrum of the sample reflected the crystal phase structure of the catalyst. The peaks at 2θ = 28.41°, 24.23°, 31.63°, and 40.5° corresponded to K_2_O, and the peaks at 2θ = 49.41° corresponded to characteristic of K_2_CO_3_ (JCPDS no: 77-2176 and 87-0730). As the preparation temperature is increased, the intensity of the characteristic peaks of K_2_O and K_2_CO_3_ gradually enhanced. The pyrolyzed durian shell, as shown in the Figure 1, showed a broad peak at 10–30° as well as a relatively weak peak in the region of 35–50°, assigned to amorphous carbon, while the peak at 2θ = 26.2° showed the presence of graphite.

**Figure 2:**
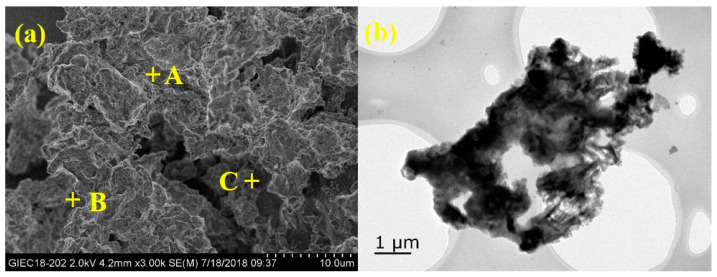
The SEM image (**a**) and TEM image (**b**) of the catalyst. A, B and C were the typical points to determine the elemental contents.

**Figure 3:**
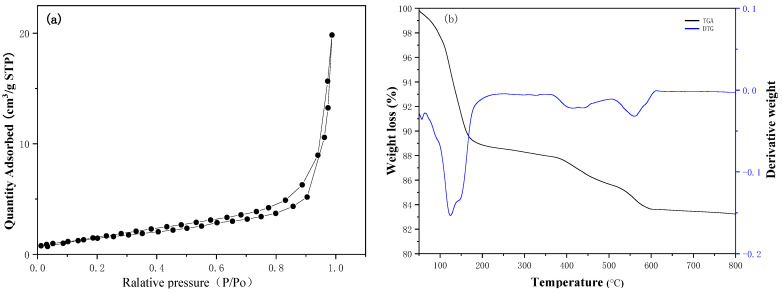
The N_2_ adsorption–desorption curve of the samples (**a**) and the TGA measurement of the DN-350 catalyst (**b**).

As shown in Table 1, the S_BET_ and pore volumes of the durian shell before treatment were estimated to be 20.25 m^2^/g and 0.057 cm^3^/g. After combustion, the S_BET_ (from 20.25 m^2^/g to 28.30 m^2^/g) as well as pore volume (from 0.057 cm^3^/g to 0.076 cm^3^/g) increased. Once combustion temperature exceeded 350 °C, the specific surface area decreased. At low combustion temperature, part of the charcoal in the durian shell burned or decomposed to form the pore structure (as shown in Figure 2a), while at high combustion temperature, the charcoal in the durian shell burned out, and the residue was mostly inorganic salts, while the S_BET_ was small. It is worth noting that under the burning conditions with low oxygen concentration, PDN-350 had a larger specific surface area, and the pyrolytic carbon had a relatively developed pore structure. In addition, Figure 3a showed the N_2_ adsorption–desorption curve of DN-350 exhibited type-IV models as the evidence of the mesoporous structure of the material.

The basicity of the catalyst is shown in Table 1. Both the alkali strength (H < 7.2) and total alkalinity (0.8 mmol/g) of durian shells were relatively low. With the combustion temperature up to 350 °C from 0 °C, the base strength and total basicity increased significantly (from 0.8 mmol/g to 11.4 mmol/g), which could be attributed to the formation of K_2_O and K_2_CO_3_ on the surface of catalyst after calcination, in accordance of XRD results. However, further increasing the combustion temperature, the total basicity of the sample hardly changed (from 11.4 mmol/g to 11.6 mmol/g) because the carbon in the durian shell was completely burned, which is consistent with Table 1. The dramatically reduced S_BET_ was likely to be the primary cause of the decrease in the basic site of the catalyst surface.

As shown in Figure 2, the SEM and TEM images of the sample show the morphological structure of the catalyst surface and interior, respectively. A large number of mesopores and micropores re observed in the SEM images (Figure 2a), and the same results can be found in the TEM images (Figure 2b), which would accelerate the mass transfer and enlarge the reaction contact area of the reactants in the transesterification.

Three typical points (A, B and C) in Figure 2a on the surface of samples were selected in order to determine the elemental contents. And the EDX result in Table 2 was the average content of the main elements which showed that the main elements on the surface of the sample were K, C, and O, and this result confirmed the conclusion of XRD. As the combustion temperature increased, the C content gradually decreased (from 64.10% to 8.25%), while the K content increased (from 13.1% to 48.30%).

As shown in Figure 3b, the TGA curve of the sample shows the weight loss of the catalyst with continuously increasing temperature under a nitrogen atmosphere. It can be seen that the sample had a weight loss of approximately 9% around 150 °C, mainly due to the removal of moisture in the catalyst. Further weight loss was caused by the decomposition of K_2_CO_3_.

**Figure 4:**
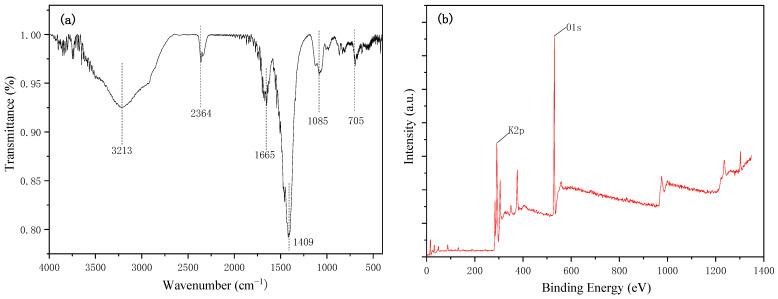
The FT-IR results (**a**) and XPS analysis of the DN-350 catalyst (**b**).

As can be seen from Figure 4a, the FT-IR spectra of the sample indicated the peak at 1655 cm^−1^, 1390 cm^−1^, and 980 cm^−1^, which could be attributed to CO stretching and bending vibrations for its carbonate composition. The peaks around 3431 cm^−1^ and 687 cm^−1^ were assigned to the stretching vibration of the K-O. The above results indicate the presence of K_2_O and K_2_CO_3_, which coincided with the results of XRD.

XPS analysis was conducted to further detect the element state on the surface of catalyst. As shown in Figure 4b, the dominating components were C (40.33%), O (32.27%), and K (12.23%). It is worth noting that two binding energies of K 2p can be observed to be 291.6 eV and 294.2 eV, suggesting the coexistence of both K_2_O and K_2_CO_3_ on the surface of catalysts, which was entirely consistent with the previous results.

### 3.2. Influence of Preparation Conditions on the Transesterification Reaction

**Figure 5:**
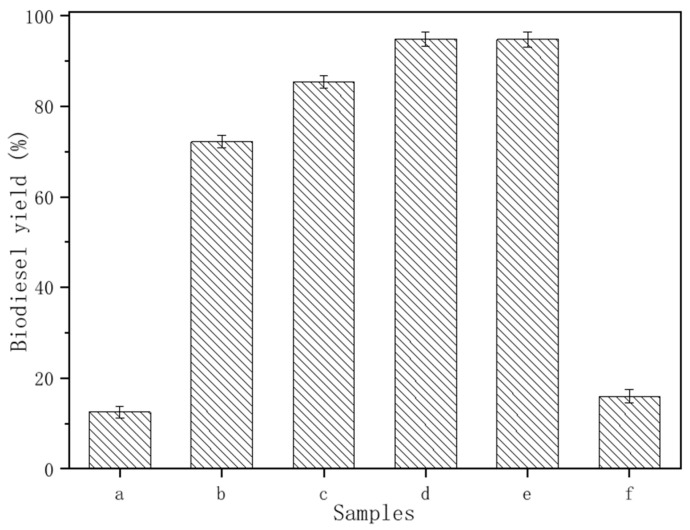
Biodiesel yield of different catalysts: (a) DN, (b) DN-250, (c) DN-300, (d) DN-350, (e) DN-400, and (f) PDN-350.

From the data in Figure 5, it can be found that the untreated durian shell (DN) had poor activity (the surface has no active substance). As the treatment temperature went up to 250 °C, the biodiesel yield of DN-250 was 72%. This is due to the formation of potassium oxides and potassium salts on the surface of the durian shell by calcination, just as shown in the XRD and XPS results. With the temperature rising to 350 °C, the activity of the catalyst was significantly improved, with an achieved biodiesel yield of 94.8%. This is because more potassium compounds were formed, resulting in its increased activity according to the XRD and EDX data. However, once the treatment temperature was beyond 400 °C, there was no obvious change of catalytic activity. Judged by the data of XRD and EDX, the higher the temperature, the more potassium oxide formed but the less carbon formed. Due to the fact that the oxides and potassium salts of potassium continued to increase at high temperature, the carbon serving as a carrier was reduced, resulting in a rapid decrease in the specific surface area and a decrease in alkalinity per unit (as listed in Table 1). It is worth noting that the PDN-350 catalyst prepared under a low oxygen level did not exhibit good activity due to the excessive content of carbon. It can be seen that moderate amount of carbon, which acts as a carrier, ensured the activity of the catalyst.

### 3.3. Influence of Reaction Conditions on the Transesterification Reaction

**Figure 6:**
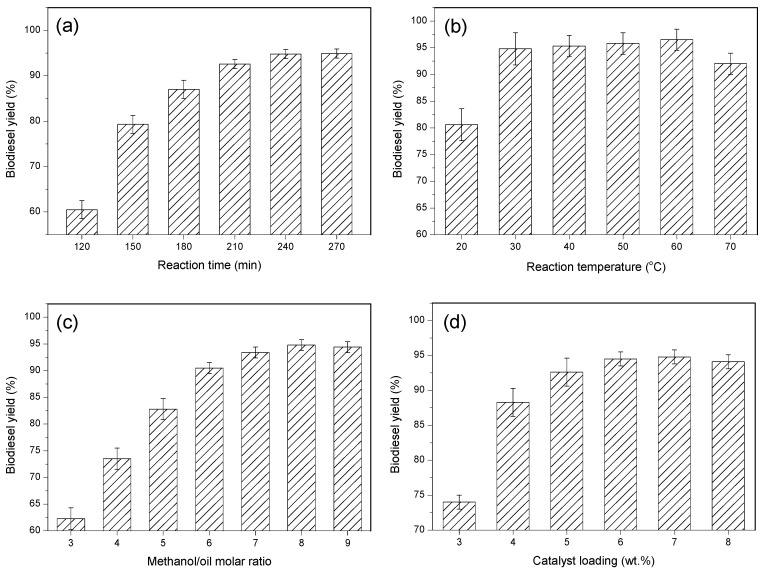
Influence of reaction conditions on the transesterification reaction: (**a**) reaction time, (**b**) reaction temperature, (**c**) methanol/oil molar ratio and (**d**) catalyst loading.

Reaction time is an important factor that cannot be ignored to affect the biodiesel yield. The influence of reaction time on yield was examined at room temperature with the previously optimal parameters. The results showed that (as shown in Figure 6a) the optimum reaction time was 2.5 h, and less than that, the yield was relatively low; higher than that, the yield did not yet increase significantly.

Reaction temperature is an important factor that determines the reaction rate. The reaction temperature for the biodiesel synthesis was set to 20 °C to 70 °C, with other conditions (reaction time of 4 h, methanol/oil molar ratio of 8:1, and catalyst amount of 8 wt.%) remaining unchanged. As shown in Figure 6b, with the reaction temperature increasing from 20 °C to 30 °C, the catalyst exhibited strong activity, and the biodiesel yield reached 94.8% at room temperature. When the temperature continued to rise (65 °C), the biodiesel yield reached 95.5%, whereas further increasing the temperature could not accelerate the reaction rate since liquid methanol evaporated into the gas phase. Considering that the catalyst had high activity under room temperature, 30 °C was the optimum temperature for reaction.

Methanol/oil molar ratio is also an important factor affecting the yield of biodiesel. At room temperature, the ratio of methanol to palm oil was varied from 3:1 to 11:1. As can be seen in Figure 6c, biodiesel yield was 60.3% when the methanol/oil molar ratio was 3:1. As the methanol/oil molar ratio increased to 8:1, the yield of biodiesel also reached 94.8%, which is the maximum. Further increasing methanol/oil molar ratio may cause the reaction to proceed in a direction unfavorable to biodiesel yield due to its reversible reaction. In other words, a methanol/oil molar ratio of 8:1 was the best value in this experiment.

The amount of catalyst also affects the yield of biodiesel. Experiments were conducted under room temperature with catalyst loading range from 4 wt.% to 10 wt.%. As shown in Figure 6d, when the catalyst loading was 4 wt.%, the biodiesel yield was only 65.7% due to its insufficient basic site. With the catalyst loading increasing from 4 to 8 wt.%, the yield reached a maximum value of 94.8%. However, further increasing the dosage, the yield was no longer enhanced due to higher viscosity of reaction system, which obstructed the mass transfer.

### 3.4. Reusability of the Catalyst

**Figure 7:**
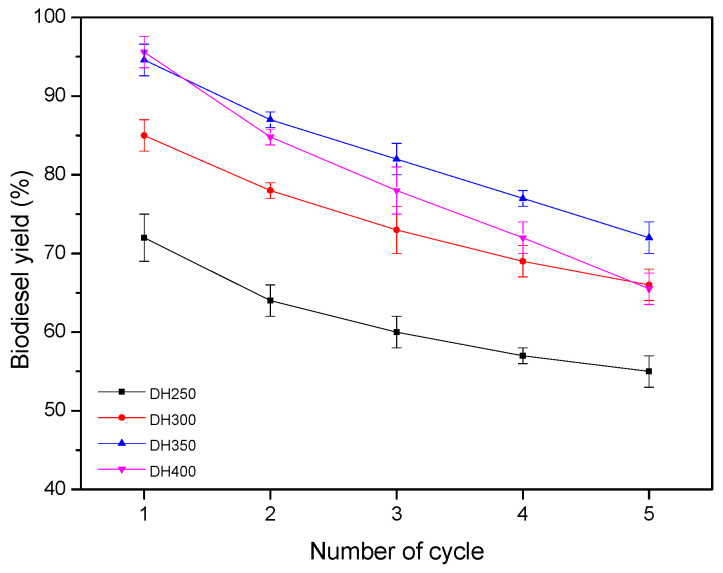
Reusability study of the catalysts.

In addition to catalyst activity, stability experiments of different catalysts were conducted, including DN250, DN300, DN350, and DN400. The reusability of the catalyst was tested by continuously repeating for five cycles under optimal conditions. As shown in Figure 7, compared with DN400, the activity of DN250, DN300, and DN350 decreased relatively slowly during the five cycles. According to the previous characterization results, it can be inferred that the presence of char in the biomass improved the stability of the catalyst. According to the EDX analysis before and after four catalysts (Table 3), it can be seen that the K concentrations of the four catalysts were significantly decreased. The main reason for the decreased activity was the loss of K on its surface. Compared with Table 3, the catalyst exhibited a high content of carbon and K. Therefore, the carbon in the catalyst prepared directly from the potassium-containing biomass can slow down the loss of active substance to some extent.

## 4. Conclusions

In summary, durian shell, as a biomass waste, can be used as a catalyst after simple treatment without functionalization for biodiesel production. Benefitting from the high content of K and Ca, the catalysts exhibited good performance in transesterification of palm oil with methanol. Considering that its preparation is simple, chemical treatment and high temperature are not required, and the precursor is a natural waste, our catalyst has good application prospects. The transesterification was carried out under room temperature with the following conditions: methanol/oil molar ratio of 1:8, reaction time of 3 h, catalyst dosage of 4 wt.%, and a maximum yield of 94.8%. The reusability study showed that the catalyst prepared at 350 °C showed better stability than the other catalyst, and its optimum yield was 70% after repeated use for five times. This is mainly because the incomplete combustion of the remaining carbon slowed down the loss of K to some extent. As a biomass waste, the catalyst has the advantages of no cost, easy preparation, high efficiency, and economic and environmental friendliness, which contribute to its status as a promising alternative for the “green catalyst” in industrial-scale, cost-efficient biodiesel production.

## Figures and Tables

**Table 1:** Surface area results and basicity properties of catalysts.

Catalyst	SBET (m^2^/g)	Pore Volume (cm^3^/g)	Basic Strength (H_)	Total Basicity (mmol/g)
DN	20.25	0.057	H_ < 7.2	0.8
DN-250	23.35	0.065	9.8 < H_ < 15.0	6.6
DN-300	28.30	0.076	15.0 < H_ < 18.4	9.4
DN-350	24.36	0.068	15.0 < H_ < 18.4	11.4
DN-400	15.91	0.045	15.0 < H_ < 18.4	11.6
PDN-350	44.71	0.114	7.2 < H_ < 9.8	1.6

**Table 2:** EDX analysis of DN-350 catalyst.

Sample	Elements Content (wt.%)						
	C	K	O	Si	Ca	Mg	Other
DN	64.10	13.1	18.79	0.81	2.10	0.50	0.60
DN-250	24.23	30.21	41.5	1.22	0.88	1.43	0.53
DN-300	16.26	37.14	40.93	1.57	1.01	2.61	0.48
DN-350	11.25	42.42	39.26	2.04	1.21	3.2	0.62
DN-400	8.25	48.30	34.35	2.87	1.51	4.06	0.66
PDN-350	61.76	23.22	10.12	2.64	1.11	0.53	0.62

**Table 3:** EDX analysis of the reused catalysts.

Catalyst	Element Content (wt.%)						
	C	K	O	Ca	Si	Mg	Others
DN-250	40.23	25.21	30.06	1.33	0.95	1.66	0.56
DN-300	36.26	30.14	27.47	1.57	1.21	2.81	0.54
DN-350	31.95	34.42	26.26	2.04	1.21	3.44	0.68
DN-400	35.37	29.28	25.35	3.27	1.88	4.16	0.69

## Data Availability

Not applicable.
